# Engineering a SERS Sensing Nanoplatform with Self-Sterilization for Undifferentiated and Rapid Detection of Bacteria

**DOI:** 10.3390/bios13010075

**Published:** 2023-01-01

**Authors:** Jun Cao, Wei Zhu, Ji Zhou, Bai-Chuan Zhao, Yao-Yu Pan, Yong Ye, Ai-Guo Shen

**Affiliations:** 1 College of Chemistry and Chemical Engineering, Wuhan Textile University, Wuhan 430200, China; 2 School of Chemistry and Chemical Engineering, Hubei University, Wuhan 430062, China; 3 Research Center of Graphic Communication, Printing and Packaging, Wuhan University, Wuhan 430079, China

**Keywords:** SERS nanoplatform, bacterial detection, CTAB, self-sterilizing

## Abstract

The development of a convenient, sensitive, rapid and self-sterilizing biosensor for microbial detection is important for the prevention and control of foodborne diseases. Herein, we designed a surface-enhanced Raman scattering (SERS) sensing nanoplatform based on a capture–enrichment–enhancement strategy to detect bacteria. The gold−Azo@silver−cetyltrimethylammonium bromide (Au−Azo@Ag−CTAB) SERS nanotags were obtained by optimizing the synthesis process conditions. The results showed that the modification of CTAB enabled the nanotags to bind to different bacteria electrostatically. This SERS sensing nanoplatform was demonstrated to be fast (15 min), accurate and sensitive (limit of detection (LOD): 300 and 400 CFU/mL for *E. coli* and *S. aureus*, respectively). Of note, the excellent endogenous antibacterial activity of CTAB allowed the complete inactivation of bacteria after the assay process, thus effectively avoiding secondary contamination.

## 1. Introduction

Foodborne illness caused by microorganisms has become a serious public health issue around the globe [1,2,3,4,5,6,7]. Pathogenic bacteria even with a low infectious dose in food and water can cause many serious and even fatal diseases due to their rapid reproductive capacity [8]. Therefore, sensitive and accurate detection of pathogens can be an effective measure for the prevention and control of foodborne diseases. However, traditional bacterial culture methods, polymerase chain reaction (PCR) and enzyme-linked immunosorbent assays (ELISA) often encountered some common problems when used for bacterial detection, including long procedural time, expensive reagents, complex sample preparation and specialized operational skills [9,10,11]. There is thus an urgent need to develop a convenient, rapid, low-cost and sensitive method for microbial detection. In addition, it is crucial to effectively inactivate the bacteria in the sample in a timely manner after the detection process, because the surviving pathogenic bacteria can continue to multiply and may cause additional contamination and pose a serious threat to public health [12,13]. Therefore, it is of significance to develop a biosensor that integrates bacterial detection and microbial elimination to respond to public health emergencies caused by microbes.

In recent years, Au-based SERS spectroscopy has received widespread attention in the detection of pathogenic bacteria because of its high sensitivity, high spectral resolution, rapid acquisition rate and spectral fingerprinting [14,15,16,17,18]. Nevertheless, conventional SERS probes usually employ direct attachment of Raman reporter molecules to the surface of probes [19]. These reporter molecules are unstable in complex environments and even easily separated from the SERS substrates [20]. It is shown that placing the Raman reporter molecules between the core and the shell of the SERS nanotags can not only effectively prevent its leakage and avoid the interference of the reporter molecules by the complex external environment, but also help to improve the sensitivity of the detection [21,22]. Recently, specific SERS probes with photothermal activity have been developed and used for the quantitative analysis and elimination of specific bacteria [23,24]. However, this type of bacterial inactivation requires the use of an external laser light source, which increases the complexity of the experimental operations. In addition, although specific bacterial recognition elements such as antibodies have good binding affinity for target bacteria [25,26], these probes lack universality and thus it is necessary to redesign and prepare SERS probes for different target bacteria. Therefore, it is desirable to develop universal SERS probes with self-sterilization capability for microbial detection.

**Figure 1:**
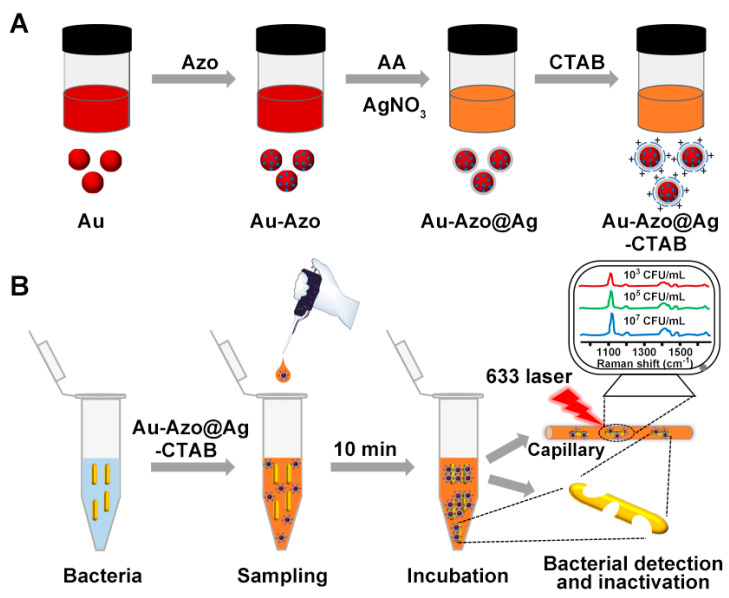
Schemes illustrating the (**A**) synthesis of Au−Azo@Ag−CTAB SERS nanotags and (**B**) the process of bacterial detection using the nanotags.

Here, we proposed a universal SERS nanotag based on the electrostatic attraction between positively charged CTAB and bacteria (negative charge at physiological pH conditions). To avoid the intrinsic fluorescence interference of conventional Raman reporter molecules (e.g., rhodamine), based on the Azo-enhanced Raman scattering strategy [27,28], Azo with an extended conjugated unit and a highly symmetric molecular structure was selected as a Raman reporter. Azo reporters were modified onto nano Au by Au-S bonding, followed by the growth of Ag shells and surface functionalization with CTAB to obtain Au−Azo@Ag−CTAB nanotags (Figure 1A). The nanotags were incubated with bacteria for 10 min and the samples were collected in glass capillary for direct detection (Figure 1B). Benefiting from the aggregation effect induced by the electrostatic interactions [29,30], the nanotags enabled the quantitative detection of *E. coli* and *S. aureus*. Furthermore, since CTAB leads to the destruction of the bacterial surface structure, resulting in significant collapse of the bacteria and leakage of the cytoplasm [31,32], the SERS nanotags exhibited excellent intrinsic bactericidal activity, thus avoiding secondary contamination after bacterial detection.

## 2. Materials and Methods

### 2.1. Materials

Chloroauric acid tetrahydrate (HAuCl_4_∙4H_2_O), sodium citrate (C_6_H_5_Na_3_O_7_∙2H_2_O), silver nitrate (AgNO_3_), ascorbic acid (AA), dimethyl sulfoxide (DMSO), sodium chloride (NaCl), and CTAB were purchased from Aladdin Bio-Chem Technology Co., Ltd. (Shanghai, China). Azo reporters (Figure S1) were kindly provided from Prof. Tingjuan Gao’s group (Central China Normal University, China). *E. coli* (ATCC 8099) and *S. aureus* (ATCC 25923) were supplied by China Center for Culture Collection (Wuhan, China). Tryptone and yeast extract were purchased from Sigma-Aldrich (Saint Louis, MO, USA). Lysogeny broth (LB) was prepared by dissolving tryptone (10 g/L), yeast extract (5 g/L), and NaCl (5 g/L) into the phosphate buffered saline solution (PBS, 10 mM, pH = 7.4), followed by autoclaving at 121 °C in an autoclave (LDZX-50L, Shanghai shenan, China). LB solid medium was prepared by mixing the LB with agar (1.5 g/L, Sigma-Aldrich). Deionized water was drawn from a Millipore Direct Q8 system with a resistivity of 18.2 MΩ cm (Millipore advantage A10 system, Merck, Germany). All chemicals were analytically pure and used without any further purification unless otherwise stated.

### 2.2. Preparation of Au Nanoparticles (NPs)

Au NPs were synthesized by the classical reduction method of sodium citrate [33]. 100 mL of deionized water was added to a flask washed with aqua regia, lye and deionized water, respectively, and then the flask was heated in an oil bath at 130 °C under stirring (1500 rpm). After boiling, 1 mL of HAuCl_4_ solution (0.29 mM) was added and heating continued for 10 min, followed by quick addition of 1.2 mL of sodium citrate solution (38.8 mM). Au NP was obtained after continuing stirring for 30 min. After cooling naturally to room temperature, the Au NP solution was stored in a refrigerator at 4 °C.

### 2.3. Preparation of Au−Azo NPs

A total of 5 mL of the above Au NP solution was added into glass vials, followed by the addition of different volumes of Azo reporter solution (1, 2, 5, 10 and 15 µL, 1mM) pre-dissolved in DMSO, respectively. The mixed solution was stirred (800 rpm) at room temperature for 4 h and then washed three times by centrifugation (3000 rpm, 30 min) using a benchtop centrifuge (TG16-11, Pingfan Instrument & Meter Co. Ltd., Changsha, China). Finally, the different Au−Azo NP solutions were stored in the refrigerator at 4 °C for further use.

### 2.4. Preparation of Au−Azo@Ag NPs

A total of 5 mL of the as-prepared Au−Azo NP solution was added into a flask, and then 200 μL of sodium citrate solution (4.8 mM) was added and the mixed solution was stirred for 5 min (800 rpm), followed by rapid addition of 50 μL of AA solution (0.1 M). Then, different volumes of AgNO_3_ solutions (0.01 M, 25, 50, 75, 100 μL) were added into the flasks, respectively. After continuing stirring for 2 h at room temperature, the reaction solution was washed three times by centrifugation (3000 rpm, 30 min) to remove the free Ag ions. Finally, the Au−Azo@Ag NPs were resuspended with deionized water and stored in the refrigerator at 4 °C for further use.

### 2.5. Preparation of Au−Azo@Ag−CTAB NPs

A total of 10 mL of the prepared Au−Azo@Ag NP solution was added into a flask. Then, different volumes of CTAB solutions (0.01 M, 50, 100, 200 μL) were added and the mixed solution was stirred for 5 min (800 rpm). The reaction solution was left to settle overnight at room temperature, followed by washing three times by centrifugation (3000 r/min, 30 min) to remove free CTAB. Finally, the Au−Azo@Ag−CTAB NPs were resuspended with deionized water and stored in the refrigerator at 4 °C.

### 2.6. Ultraviolet-Visible (UV-vis) Spectral Characterization

The UV-vis absorbance of the product in each step was recorded using a UV-3600 spectrophotometer (Shimadzu). UV-vis spectroscopic characterization provides a reference for NP stability. The UV-vis spectra were obtained as follows: after baseline calibration, the sample solution was added to the cuvette and then scanned in the selected wavelength range (300–800 nm).

### 2.7. Dynamic Light Scattering (DLS) and Zeta Potential Characterization

The hydrated size and zeta potential of NPs were determined by using the Malvern particle sizer (ZS90, Malvern). The DLS characterization showed the size distribution of NPs, while the zeta potential characterization showed the surface electrical properties of NPs. The details are as follows: the NP solution was added to a specific sample cell (size: DTS0012, potential: DTS1070) and then the sample solution was subsequently detected by the corresponding mode.

### 2.8. Field Emission Scanning Electron Microscopy (FE-SEM) Characterization

The morphology of NPs, Au−Azo@Ag−CTAB anchored *E. coli* and Au−Azo@Ag−CTAB anchored *S. aureus* was characterized by FE-SEM (Zeiss SIGMA, Carl Zeiss AG, Jena, Germany). The specific experimental method was as follows: the appropriate concentration of sample solution was dropped onto a conductive single-crystal silicon wafer. After drying naturally, the samples sprayed with gold were characterized morphologically using an Inlens detector. The test voltage was 10 KV.

### 2.9. Raman Spectroscopy Characterization

To optimize the addition volume of Azo reporters, Ag ions and CTAB, the Au−Azo NPs, Au−Azo@Ag NPs and Au−Azo@Ag−CTAB NPs were characterized by Raman spectroscopy (InVia, Renishaw, Wotton-under-Edge, UK), respectively. The experimental procedure was as follows: a certain volume of sample solution was aspirated with a glass capillary tube (5 mm diameter), and then the capillary tube was placed under a microscope (20× objective) and the sample stage was adjusted so that the capillary tube was in focus under the laser, followed by Raman signal acquisition. Acquisition conditions: excitation wavelength of 633 nm, laser power of 10 mW, integration time of 1 s, and accumulation of 1 time. The Raman spectra were acquired in the spectral range from 950 to 1620 cm^−1^. The initial Raman spectrum in this study was calibrated and smoothed by WIRE 3.4 software (intelligent fitting, polynomial order of 10, noise tolerance of 1.50), and the peak intensity at 1120 cm^−1^ (Ph-N) was used for quantitative analysis [28].

### 2.10. Bacterial Culture

The lyophilized powder of *E. coli* (ATCC 8099) and *S. aureus* (ATCC 25923) was transferred into LB liquid medium (5 mL) respectively, followed by incubation (37 °C, 200 rpm) in a constant temperature shaking incubator (PTQZ-6, Changzhou Putian Instrument Manufacturing Co., Ltd., Changzhou, China) until the bacterial concentration reached 1 × 10^8^ CFU/mL. Afterward, the two bacteria solutions were collected by centrifugation (3000 rpm, 10 min) and washed with PBS three times. Prior to the test of the bacterial sample, the concentrations of bacteria suspended in PBS were determined by spread plate method.

### 2.11. Bacterial Quantification Using Au−Azo@Ag−CTAB NPs

The two bacterial solutions (*S. aureus* and *E. coli*) were diluted separately with PBS to a concentration of 1 × 10^7^ CFU/mL according to the results of plate counting. Then the diluted bacterial solution was used as mother liquor to prepare four groups of bacterial solutions with different concentrations (1 × 10^6^ CFU/mL, 1 × 10^5^ CFU/mL, 1 × 10^4^ CFU/mL and 1 × 10^3^ CFU/mL). Afterwards, 20 μL of Au−Azo@Ag−CTAB NP solution was added to 200 μL of different concentrations of bacterial solution, respectively. The mixture was shaken for 10 min and then aspirated with a capillary tube and placed under a microscope (10× objective, NA = 0.4) for Raman spectroscopy. The test conditions were the same as described in Section 2.9.

### 2.12. Bacterial Detection and Inactivation in Tap Water

The *S. aureus* and *E. coli* with unknown concentrations were added to the tap water and shaken thoroughly for 10 min, respectively. Then 100 μL of the above bacterial solution was blended with 20 μL of Au−Azo@Ag−CTAB NP solution. The Raman spectra were acquired according to the method constructed in Section 2.11. The accuracy of our method in detecting bacteria in real samples was evaluated by comparing it with the culturing results. To verify the antibacterial ability of Au−Azo@Ag−CTAB nanotags, the bacteria/Au−Azo@Ag−CTAB nanotag mixture was plated on a solid LB medium and incubated for 24 h at 37 °C, followed by counting the number of the bacterial colonies. The bacteria without any treatment were acted as control.

## 3. Results

### 3.1. Synthesis and Characterization of Au−Azo@Ag−CTAB NPs

During the preparation of the Au−Azo@Ag−CTAB NPs, the addition amount of the Azo signal molecules, AgNO_3_ and CTAB has an important impact on the SERS signal strength and stability of the NPs, and thus it is necessary to optimize these parameters.

#### 3.1.1. Optimization of Synthesis Conditions of Au NPs

**Figure 2:**
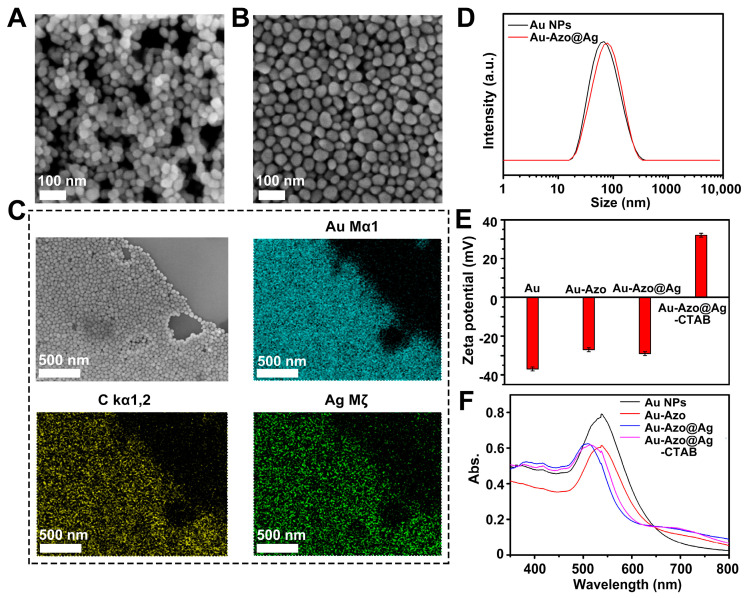
(**A**) SEM images of Au NPs. (**B**) SEM images of Au−Azo@Ag−CTAB NPs. (**C**) Energy dispersive spectroscopy (EDS) mapping images of Au−Azo@Ag−CTAB NPs (SEM). (**D**) Hydrated particle size of Au and Au−Azo@Ag NPs. (**E**) Zeta potential of different NPs. (**F**) UV-vis spectra of different NPs.

The Au NPs were first prepared by the classical sodium citrate reduction. It has been reported that the SERS enhancement effect of the Au NPs was related to its size [34]. When the size of the Au NPs increased, the collective oscillation amplitude of free electrons on its surface decreased and the absorption band of surface plasmon resonance broadened, leading to a weaker SERS enhancement effect. However, the SERS enhancement effect was also weak for the small-sized nano Au because of the low light absorption cross section and the low number of excited electrons on the surface. Combining the above factors, the Au NPs with a size of 45 nm were successfully prepared (Figure 2A).

#### 3.1.2. Optimization of Synthesis Conditions of Au−Azo NPs

Azo reporters can be modified onto the nano Au through Au-S bonds. The addition volume of Azo reporters was optimized, and the products were marked as Au−Azo−1 to Au−Azo−5 in the order of volume from small to large. The UV-vis spectrum of each product is shown in Figure S2A. With the increase in the addition volume of Azo reporters, the peak intensity of the product at 520 nm gradually reduced, in which the Au−Azo−5 NP solution showed significant sedimentation (as shown in the inset), indicating that the stability of the NPs decreased gradually. In addition, the products were further characterized by Raman spectroscopy. As shown in Figure S2B, compared with other NPs, Au−Azo−4 showed the best SERS enhancement effect, in which the Raman signal at 1120 cm^−1^ can be attributed to the stretching vibration of the Ph-N bond in Azo. However, the Au−Azo−4 NPs presented an obvious UV-vis absorption peak near 700 nm, which indicated a certain degree of aggregation of the NPs. Collectively, the Au−Azo−3 NPs were selected for subsequent experiments.

#### 3.1.3. Optimization of Synthesis Conditions of Au−Azo@Ag NPs

Similarly, the addition volume of AgNO_3_ was optimized and the products were labeled as Au−Azo@Ag−1 to Au−Azo@Ag−4. As shown in Figure S3A, with the increase in the addition volume of AgNO_3_, the UV-vis absorption peak of the Au−Azo@Ag NPs was gradually blue-shifted. The Au−Azo@Ag−3/−4 NP solution presented an obvious absorption peak at 620 nm, and the color of the solution was turbid (as shown in the inset), indicating a certain degree of aggregation of the NPs. In addition, the products were also characterized by Raman spectroscopy (Figure S3B). Based on the stability and SERS enhancement effect of the Au−Azo@Ag NPs, the Au−Azo@Ag−2 NPs were selected for subsequent experiments.

#### 3.1.4. Optimization of Synthesis Conditions of Au−Azo@Ag−CTAB NPs

The addition volume of CTAB was optimized and the products were labeled as Au−Azo@Ag−CTAB−1 to Au−Azo@Ag−CTAB−3. Because the surface of the Au−Azo@Ag NPs presented a negative charge, CTAB with a positive charge can be modified to the surface of the Au−Azo@Ag NPs by electrostatic action. Figure S4 showed the UV-vis spectra of the Au−Azo@Ag−CTAB NPs with different surface charge densities. With the increase in the CTAB addition volume, the Au−Azo@Ag−CTAB NPs presented a weaker absorption peak at 700 nm, indicating better dispersion stability. The Au−Azo@Ag−CTAB−3 NPs were selected as the optimal SERS nanotags. For the convenience of naming and description, it was uniformly called the Au−Azo@Ag−CTAB NPs in the subsequent experiment.

#### 3.1.5. Characterization of Au−Azo@Ag−CTAB NPs

The Au−Azo@Ag−CTAB NPs with a size of about 55 nm were successfully synthesized step-by-step (Figure 2B). The results of the EDS-mapping also demonstrated the successful synthesis of the Au−Azo@Ag−CTAB NPs (Figure 2C). In addition, the hydrated size of the Au−Azo@Ag NPs was significantly larger than the Au NPs, indicating the successful growth of the Ag shells onto the nano Au (Figure 2D). The results from zeta potential characterization demonstrated that the CTAB (positive charge) was successfully modified on the surface of the Au−Azo@Ag NPs (Figure 2E). The UV-vis spectrum of each product is shown in Figure 2F. The blue shift of the absorption peak wavelength of the Au−Azo@Ag NPs with respect to the Au NPs indicated the successful formation of the Ag shells.

### 3.2. Ability of Au−Azo@Ag−CTAB NPs to Capture, Detect and Inactivate Bacteria

**Figure 3:**
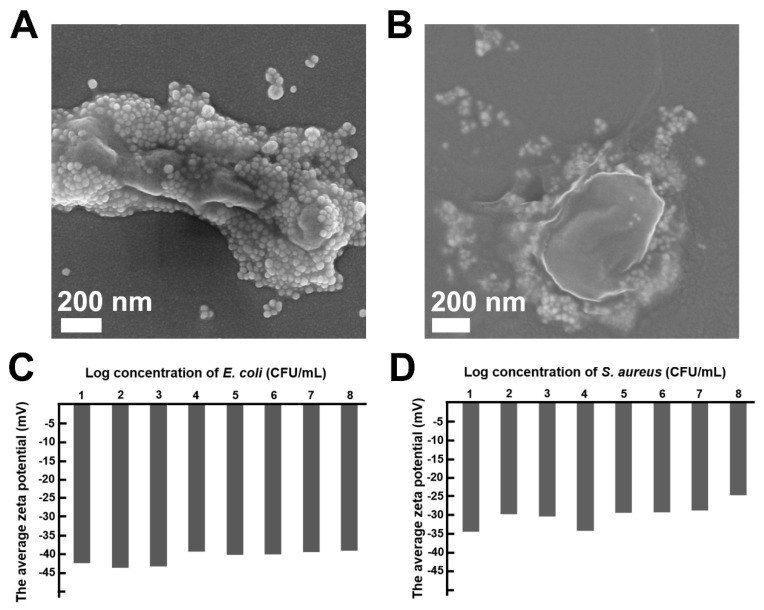
SEM images of (**A**) *E. coli* and (**B**) *S. aureus* anchored by Au−Azo@Ag−CTAB NPs. Surface potential of (**C**) *E. coli* and (**D**) *S. aureus* at different concentrations.

After mixing the positively charged Au−Azo@Ag−CTAB NPs with bacteria (*S. aureus* or *E. coli*), the bacteria can induce the aggregation of the NPs through electrostatic effect, thereby enhancing the SERS signal of the detection system [35]. In order to verify the detection mechanism of this study, the combination of nanotags and bacteria was characterized by SEM. As shown in Figure 3A,B, a large number of NPs were anchored onto the surface of *E. coli* and *S. aureus*, respectively, and the density of the nanotags on the surface of *E. coli* was slightly higher than that of *S. aureus*, which may be due to the relatively lower surface potential of *E. coli* (Figure 3C,D).

**Figure 4:**
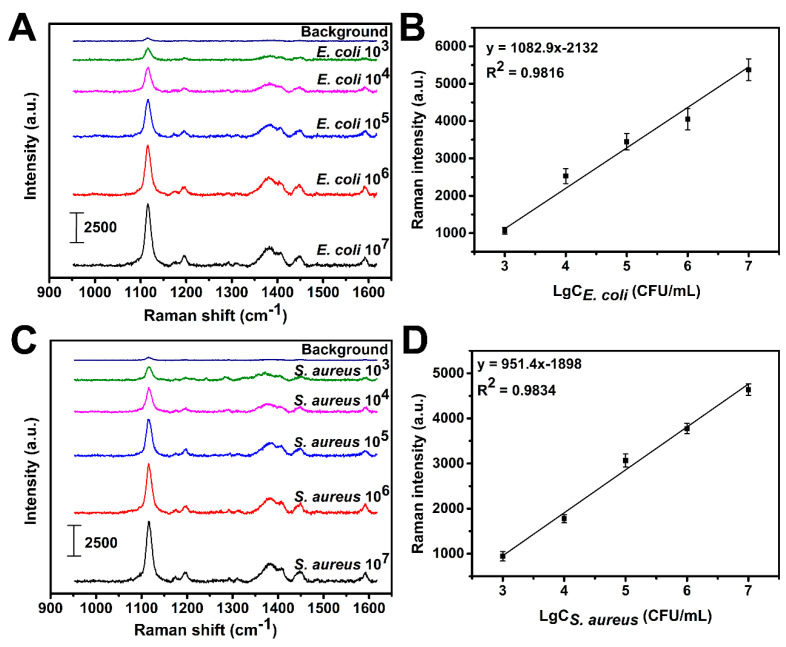
Raman spectra of Au−Azo@Ag−CTAB nanotags after co-incubation with different concentrations of (**A**) *E. coli* and (**C**) *S. aureus*. Calibration curve between the Raman signal intensity with the concentration of (**B**) *E. coli* and (**D**) *S. aureus*.

To investigate the feasibility of this method for bacterial quantification analysis, different concentrations of *E. coli* and *S. aureus* were mixed with the Au−Azo@Ag−CTAB NPs, respectively, and then determined by Raman spectroscopy. As shown in Figure 4A, within the concentration range from 10^3^ to 10^7^ CFU/mL, the Raman intensity at 1120 cm^−1^ gradually enhanced with the increase in *E. coli* concentration. There was a good linear relationship between the Raman intensity and the logarithm of bacterial concentration (Figure 4B), and the calibration curve for *E. coli* was y = 1082.9x − 2132 with R^2^ of 0.9816, achieving LOD as low as 300 CFU/mL (3 times signal/background). In addition, the relationship between the Raman intensity and the logarithm of the bacterial concentration was further explored using *S. aureus* as a model. Similar to *E. coli*, there was a positive correlation between them (Figure 4C), and the linear fitting equation was y = 951.4x − 1898 with R^2^ = 0.9834 (Figure 4D) (LOD: 400 CFU/mL). The results indicated that the Raman intensity of the nanotags prepared in this work had a good linear correlation with the logarithm of the concentration of *E. coli* and *S. aureus*, respectively. In order to avoid experimental errors, five randomly selected positions of the capillary tube containing different concentrations of bacteria/nanotags mixture were tested by Raman spectroscopy, and the results showed the reliability of the method (Figures S5 and S6).

The Au−Azo@Ag−CTAB NPs were expected to possess excellent antibacterial ability due to the ability of CTAB to disrupt the cell surface structure [31]. To verify the bactericidal performance of the Au−Azo@Ag−CTAB NPs, high concentrations (10^7^ CFU/mL) of bacteria (*S. aureus* and *E. coli*) were incubated with nanotags for 2 h, and then characterized by the spread plate method. As shown in Figure S7, the control group (without treatment) presented a large number of bacterial colonies, while no colonies of *E. coli* or *S. aureus* were observed in the Au−Azo@Ag−CTAB NPs group, demonstrating its good antimicrobial activity. Thus, the nanotags had the potential to prevent the secondary contamination of bacteria after detection.

### 3.3. Quantitative Detection and Inactivation of Bacteria in Tap Water

**Figure 5:**
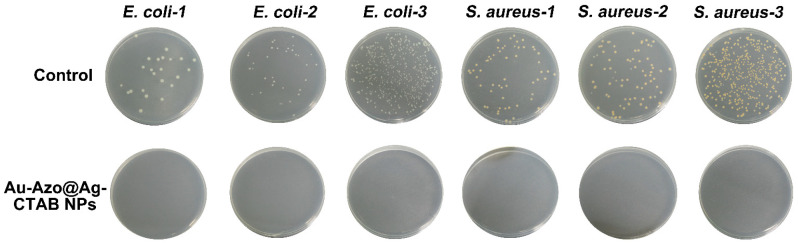
Plate colony images of *E. coli* and *S. aureus* in tap water after treatment with Au−Azo@Ag−CTAB nanotags.

Due to the increasing popularity of tap water and its use as the main source of drinking water, the detection of microorganisms in tap water is the key to ensure the safety of drinking water. In order to verify the feasibility of this method in the detection of bacteria in tap water, the method of spiked bacteria in tap water was employed. Six groups of bacterial sample solutions with unknown concentrations (*S. aureus* and *E. coli*) were mixed with the Au−Azo@Ag−CTAB nanotags, respectively, and then characterized by Raman spectroscopy under the same conditions as stated before. The bacterial concentration was calculated according to the calibration curve of the corresponding bacteria, respectively. As shown in Table 1, the concentrations of the *E. coli* and *S. aureus* detected by our method were comparable to that of the standard culture counting with the relative recovery ranging from 106% to 110%, which indicated that this method was suitable for the detection of microorganisms in tap water. The errors may be due to the interference from potential impurities such as particulate matters and ionic materials. Furthermore, the detected concentration of bacteria was relatively consistent for the repeated measurements with the RSD% less than 5%, demonstrating the reliability of our method for tap water monitoring. Compared to the time-consuming bacterial culture (2–3 days), the whole process of our method can be completed within 15 min, indicating the potential of point-of-care testing (POCT) in bacterial detection. The inactivation ability of the Au−Azo@Ag−CTAB nanotags to bacteria in tap water was characterized by the spread plate method. As shown in Figure 5, no bacterial colonies were found in the Au−Azo@Ag−CTAB nanotags group, which indicated that the bacteria in the tested solution have been completely inactivated, thus effectively avoiding environmental pollution.

## 4. Conclusions and Future Perspective

In this work, we designed a novel SERS sensing nanoplatform that integrated broad-spectrum bacterial detection and self-sterilization. The Au−Azo@Ag−CTAB nanotags were prepared based on the rational configuration of the recognition unit, Raman reporter and SERS substrate. The positively charged Au−Azo@Ag−CTAB nanotags were anchored on the surface of bacteria through electrostatic action so as to induce the enhancement of the SERS signal. The results showed that the proposed SERS sensing nanoplatform was sensitive, rapid and reliable for bacterial detection. The recoveries of *E. coli* ranged from 108% to 110%, and the recoveries of *S. aureus* ranged from 106% to 107%, both of which demonstrated the feasibility of this method for the quantification of bacteria in tap water. Furthermore, the microorganisms in the tested solution can be killed completely and timely without any treatment, avoiding bacteria contamination after the bacterial detection. The anti-interference capability of the biosensor, which binds bacteria by electrostatic interaction, may need further improvement. Therefore, in the future, we plan to modify the nanotags with mercaptophenylboronic acid to enhance the recognition of bacteria. This SERS sensing nanoplatform has potential applications in the POCT of medical and environmental samples for which rapid in situ testing is required.

## Abbreviation

SERSSurface-enhanced Raman scatteringAu NPsGold nanoparticlesCTABCetyltrimethylammonium bromidePBSphosphate buffered saline solutionSEMScanning electron microscopyPOCTPoint-of-care testingRSDRelative standard deviation
*S. aureus*

*Staphylococcus aureus*

*E. coli*

*Escherichia coli*
CFUColony-forming unitUV-visUltraviolet-visibleAgNO_3_Silver nitrateLODLimit of detection

## Figures and Tables

**Table 1:** Detection of the concentration of bacteria in tap water based on the culturing method and the constructed method in this work.

Sample	Culturing Result (CFU/mL)	Detected Result (CFU/mL)	Relative Recovery (%)	RSD ^1^ (%)
*E. coli*−*1*	2.40 × 10^3^	2.59 × 10^3^	108	4.2
*E. coli*−*2*	3.50 × 10^3^	3.85 × 10^3^	110	4.5
*E. coli*−*3*	4.85 × 10^4^	5.24 × 10^4^	108	4.1
*S. aureus*−*1*	5.70 × 10^3^	6.10 × 10^3^	107	4.3
*S. aureus*−*2*	7.50 × 10^3^	7.95 × 10^3^	106	4.2
*S. aureus*−*3*	3.81 × 10^4^	4.04 × 10^4^	106	4.3

^1^ Relative standard deviation (n = 3).

## Data Availability

The data presented in this study are available on request from the corresponding author.

## Supplementary Materials

The following supporting information can be downloaded at: https://www.mdpi.com/article/10.3390/bios13010075/s1, Figure S1: The molecular structure of Azo Raman reporter; Figure S2: Optimization of the addition volume of Azo signal molecules. (A) UV-vis absorption spectra of different Au−Azo NPs. (B) Raman spectra of different Au−Azo NPs; Figure S3: Optimization of the addition volume of AgNO_3_. (A) UV-vis absorption spectra of different Au−Azo@Ag NPs. (B) Raman spectra of different Au−Azo@Ag NPs; Figure S4: Optimization of the addition volume of CTAB. UV-vis absorption spectra of different Au−Azo@Ag−CTAB NPs; Figure S5: Raman spectra in five randomly selected positions of the capillary tube containing mixtures of different concentrations of *E. coli* and nanotags. A: 10^7^ CFU/mL; B: 10^6^ CFU/mL; C: 10^5^ CFU/mL; D: 10^4^ CFU/mL; E: 10^3^ CFU/mL. The relative standard deviation (RSD) of each group was less than 5%; Figure S6: Raman spectra in five randomly selected positions of the capillary tube containing mixtures of different concentrations of *S. aureus* and nanotags. A: 10^7^ CFU/mL; B: 10^6^ CFU/mL; C: 10^5^ CFU/mL; D: 10^4^ CFU/mL; E: 10^3^ CFU/mL. The relative standard deviation (RSD) of each group was less than 5%; Figure S7: *E. coli* and *S. aureus* colonies after incubated with Au−Azo@Ag−CTAB nanotags for 2 h.

## References

[1] Prakash O., Sil S., Verma T., Umapathy S. (2020). Direct Detection of Bacteria Using Positively Charged Ag/Au Bimetallic Nanoparticles: A Label-free Surface-Enhanced Raman Scattering Study Coupled with Multivariate Analysis. J. Phys. Chem. C.

[2] Cao Z., Li C., Yang X., Wang S., Zhang X., Zhao C., Xue B., Gao C., Zhou H., Yang Y. (2022). Rapid Quantitative Detection of Live Escherichia coli Based on Chronoamperometry. Biosensors.

[3] Al-Hindi R.R., Teklemariam A.D., Alharbi M.G., Alotibi I., Azhari S.A., Qadri I., Alamri T., Harakeh S., Applegate B.M., Bhunia A.k. (2022). Bacteriophage-Based Biosensors: A Platform for Detection of Foodborne Bacterial Pathogens from Food and Environment. Biosensors.

[4] Wang P., Sun Y., Li X., Wang L., Xu Y., He L., Li G. (2021). Recent Advances in Dual Recognition Based Surface Enhanced Raman Scattering for Pathogenic Bacteria Detection: A Review. Anal. Chim. Acta.

[5] Kaur K., Chelangat W., Druzhinin S.I., Karuri N., Müller M., Schönherr H. (2021). Quantitative *E. coli* Enzyme Detection in Reporter Hydrogel-Coated Paper Using a Smartphone Camera. Biosensors.

[6] Yan C., Sun Y., Yao M., Jin X., Yang Q., Wu W. (2022). pH-Responsive Nanoparticles and Automated Detection Apparatus for Dual Detection of Pathogenic Bacteria. Sens. Actuators B Chem..

[7] Angelopoulou M., Petrou P., Misiakos K., Raptis I., Kakabakos S. (2022). Simultaneous Detection of *Salmonella typhimurium* and *Escherichia coli O157:H7* in Drinking Water and Milk with Mach-Zehnder Interferometers Monolithically Integrated on Silicon Chips. Biosensors.

[8] Liu Y., Zhu W., Yuan Q., Hu J., Zhang X., Shen A. (2022). Photoreduced Ag^+^ Surrounding Single Poly(4-cyanostyrene) Nanoparticles for Undifferentiated SERS Sensing and Killing of Bacteria. Talanta.

[9] Chu H., Huang Y., Zhao Y. (2008). Silver Nanorod Arrays as a Surface-Enhanced Raman Scattering Substrate for Foodborne Pathogenic Bacteria Detection. Appl. Spectrosc..

[10] Kang D.K., Ali M.M., Zhang K., Huang S.S., Peterson E., Digman M.A., Gratton E., Zhao W. (2014). Rapid Detection of Single Bacteria in Unprocessed Blood Using Integrated Comprehensive Droplet Digital Detection. Nat. Commun..

[11] Ottesen E.A., Hong J.W., Quake S.R., Leadbetter J.R. (2006). Microfluidic Digital PCR Enables Multigene Analysis of Individual Environmental Bacteria. Science.

[12] Millenbaugh N.J., Baskin J.B., DeSilva M.N., Elliott W.R., Glickman R.D. (2015). Photothermal Killing of *Staphylococcus aureus* Using Antibody-Targeted Gold Nanoparticles. Int. J. Nanomed..

[13] Wei Z., Zhou Z., Yang M., Lin C., Zhao Z., Huang D., Chen Z., Gao J. (2011). Multifunctional Ag@Fe_2_O_3_ Yolk-Shell Nanoparticles for Simultaneous Capture, Kill, and Removal of Pathogen. J. Mater. Chem..

[14] Zhou Z., Xiao R., Cheng S., Wang S., Shi L., Wang C., Qi K., Wang S. (2021). A Universal SERS-Label Immunoassay for Pathogen Bacteria Detection Based on Fe_3_O_4_@Au-Aptamer Separation and Antibody-Protein A Orientation Recognition. Anal. Chim. Acta.

[15] Mosier-Boss P.A. (2017). Review on SERS of Bacteria. Biosensors.

[16] Laing S., Gracie K., Faulds K. (2016). Multiplex In Vitro Detection Using SERS. Chem. Soc. Rev..

[17] Zhang C., Wang C., Xiao R., Tang L., Huang J., Wu D., Liu S., Wang Y., Zhang D., Wang S. (2018). Sensitive and Specific Detection of Clinical Bacteria via Vancomycin-Modified Fe_3_O_4_@Au Nanoparticles and Aptamer-Functionalized SERS Tags. J. Mater. Chem. B.

[18] Xiang X., Feng S., Chen J., Feng J., Hou Y., Ruan Y., Weng X., Milcovich G. (2019). Gold Nanoparticles/Electrochemically Expanded Graphite Composite: A Bifunctional Platform Toward Glucose Sensing and SERS Applications. J. Electroanal. Chem..

[19] Zong S., Wang Z., Chen H., Yang J., Cui Y. (2013). Surface Enhanced Raman Scattering Traceable and Glutathione Responsive Nanocarrier for the Intracellular Drug Delivery. Anal. Chem..

[20] Hu Z., Zhou X., Duan J., Wu X., Wu J., Zhang P., Liang W., Guo J., Cai H., Sun P. (2021). Aptamer-Based Novel Ag-Coated Magnetic Recognition and SERS Nanotags with Interior Nanogap Biosensor for Ultrasensitive Detection of Protein Biomarker. Sens. Actuators B Chem..

[21] Lin S., Hasi W., Lin X., Han S., Xiang T., Liang S., Wang L. (2020). Lab-On-Capillary Platform for On-Site Quantitative SERS Analysis of Surface Contaminants Based on Au@4-MBA@Ag Core–Shell Nanorods. ACS Sens..

[22] Wang C., Wang J., Li M., Qu X., Zhang K., Rong Z., Xiao R., Wang S. (2016). A Rapid SERS Method for Label-Free Bacteria Detection Using Polyethylenimine-Modified Au-Coated Magnetic Microspheres and Au@Ag Nanoparticles. Analyst..

[23] Gao X., Yin Y., Wu H., Hao Z., Li J., Wang S., Liu Y. (2021). Integrated SERS Platform for Reliable Detection and Photothermal Elimination of Bacteria in Whole Blood Samples. Anal. Chem..

[24] Huang X., Zhang Z., Chen L., Lin Y., Zeng R., Xu J., Chen S., Zhang J., Cai H., Zhou H. (2022). Multifunctional Au Nano-Bridged Nanogap Probes as ICP-MS/SERS Dual-Signal Tags and Signal Amplifiers for Bacteria Discriminating, Quantitative Detecting and Photothermal Bactericidal Activity. Biosens. Bioelectron..

[25] Cheng D., Yu M., Fu F., Han W., Li G., Xie J., Song Y., Swihart M., Song E., Wang S. (2015). Dual Recognition Strategy for Specific and Sensitive Detection of Bacteria Using Aptamer-Coated Magnetic Beads and Antibiotic-Capped Gold Nanoclusters. Anal. Chem..

[26] Luo K., Ryu J., Seol I.H., Jeong K.B., You S.M., Kim Y.R. (2019). Paper-Based Radial Chromatographic Immunoassay for the Detection of Pathogenic Bacteria in Milk. ACS Appl. Mater. Interfaces.

[27] Yu Y., Tang Y., Chu K., Gao T., Smith Z.J. (2022). High-Resolution Low-Power Hyperspectral Line-Scan Imaging of Fast Cellular Dynamics Using Azo-Enhanced Raman Scattering Probes. J. Am. Chem. Soc..

[28] Tang Y., Zhuang Y., Zhang S., Smith Z., Li Y., Mu X., Li M., He C., Zheng X., Pan F. (2021). Azo-Enhanced Raman Scattering for Enhancing the Sensitivity and Tuning the Frequency of Molecular Vibrations. ACS Cent. Sci..

[29] Hayden S.C., Zhao G., Saha K., Phillips R.L., Li X., Miranda O.R., Rotello V.M., El-Sayed M.A., Schmidt-Krey I., Bunz U.H.F. (2012). Aggregation and Interaction of Cationic Nanoparticles on Bacterial Surfaces. J. Am. Chem. Soc..

[30] Hussain S., Lv F., Qi R., Senthilkumar T., Zhao H., Chen Y., Liu L., Wang S. (2020). Förster Resonance Energy Transfer Mediated Rapid and Synergistic Discrimination of Bacteria over Fungi Using a Cationic Conjugated Glycopolymer. ACS Appl. Bio Mater..

[31] Fernández-Grajera M., Gallardo-Moreno A.M., Luque-Agudo V., González-Martín M.L., Hierro-Oliva M. (2022). Bacterial Response to the Surface Aging of PLA Matrices Loaded with Active Compounds. Polymers.

[32] Abduraimova A., Molkenova A., Duisembekova A., Mulikova T., Kanayeva D., Atabaev T.S. (2021). Cetyltrimethylammonium Bromide (CTAB)-Loaded SiO_2_-Ag Mesoporous Nanocomposite as an Efficient Antibacterial Agent. Nanomaterials.

[33] Daniel M., Astruc D. (2004). Gold Nanoparticles:  Assembly, Supramolecular Chemistry, Quantum-Size-Related Properties, and Applications toward Biology, Catalysis, and Nanotechnology. Chem. Rev..

[34] Deeb C., Zhou X., Plain J., Wiederrecht G.P., Bachelot R., Russell M., Jain P.K. (2013). Size Dependence of the Plasmonic Near-Field Measured via Single-Nanoparticle Photoimaging. J. Phys. Chem. C.

[35] Bi L., Wang X., Cao X., Liu L., Bai C., Zheng Q., Choo J., Chen L. (2020). SERS-Active Au@Ag Core-Shell Nanorod (Au@AgNR) Tags for Ultrasensitive Bacteria Dtetion and Antibiotic-Susceptibility Testing. Talanta.

