# Supplementary material for: Engineering a SERS Sensing Nanoplatform with Self-Sterilization for Undifferentiated and Rapid Detection of Bacteria
Source: Biosensors (Basel). 2023 Jan 1;13(1):75. doi: 10.3390/bios13010075 (PMC9855742; doi:10.3390/bios13010075)
Supplement: Supplementary file 1 [file biosensors-13-00075-s001.zip › biosensors-2108537-supplementary.pdf]

Supplementary Materials

Engineering a SERS Sensing Nanoplatform with Self-Sterilization for Undifferentiated and Rapid Detection of Bacteria

Jun Cao [1], Wei Zhu [1], Ji Zhou [2], Bai-Chuan Zhao [3], Yao-Yu Pan [3], Yong Ye [2,*] and Ai-Guo Shen [1,3,*]

[1] College of Chemistry and Chemical Engineering, Wuhan Textile University, Wuhan 430200, China
[2] School of Chemistry and Chemical Engineering, Hubei University, Wuhan 430062, China
[3] Research Center of Graphic Communication, Printing and Packaging, Wuhan University, Wuhan 430079, China
* Correspondence: yeyong@hubu.edu.cn (Y.Y.); agshen@whu.edu.cn (A.-G.S.)

Table of Contents

Figure S1. The molecular structure of Azo Raman reporter.

Figure S2. Optimization of the addition volume of Azo signal molecules.

Figure S3. Optimization of the addition volume of $AgNO_3$.

Figure S4. Optimization of the addition volume of CTAB

Figure S5. Raman spectra in five randomly selected positions of the capillary tube containing mixture of different concentrations of *E. coli* and nanotags.

Figure S6. Raman spectra in five randomly selected positions of the capillary tube containing mixture of different concentrations of *S. aureus* and nanotags.

Figure S7. *E. coli* and *S. aureus* colonies after incubated with Au-Azo@Ag-CTAB nanotags for 2 h.

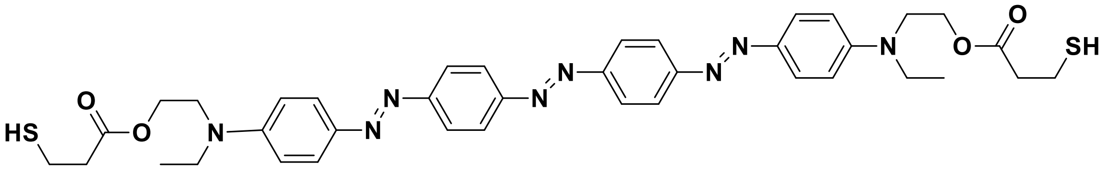

Figure S1. The molecular structure of Azo Raman reporter.

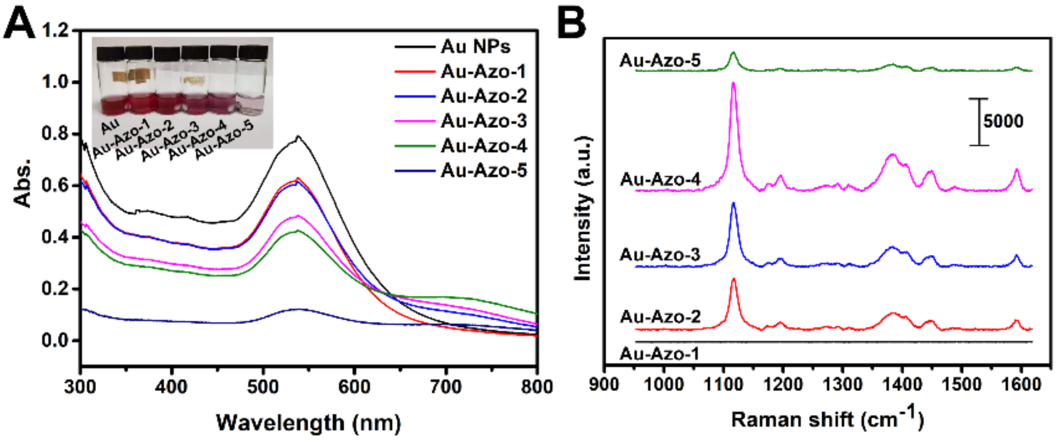

Figure S2. Optimization of the addition volume of Azo signal molecules. (A) UV-vis absorption spectra of different Au–Azo NPs. (B) Raman spectra of different Au–Azo NPs.

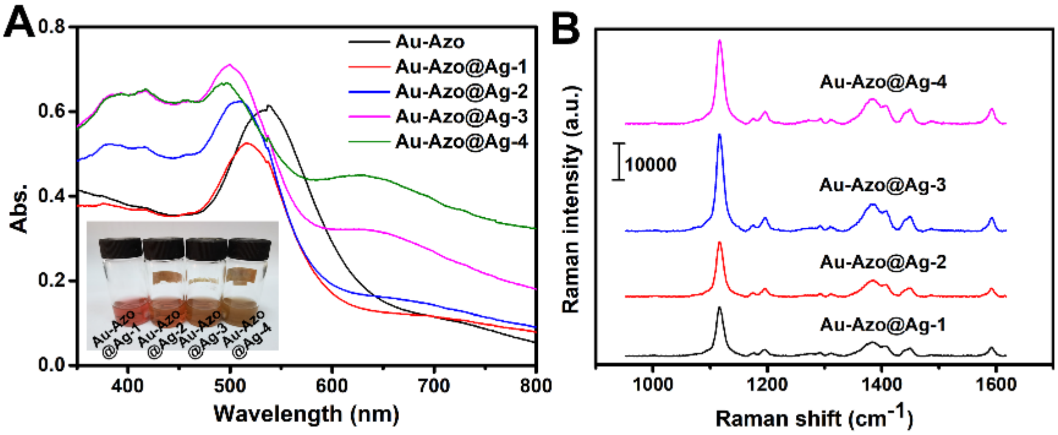

Figure S3. Optimization of the addition volume of $AgNO_3$. (A) UV-vis absorption spectra of different Au–Azo@Ag NPs. (B) Raman spectra of different Au–Azo@Ag NPs.

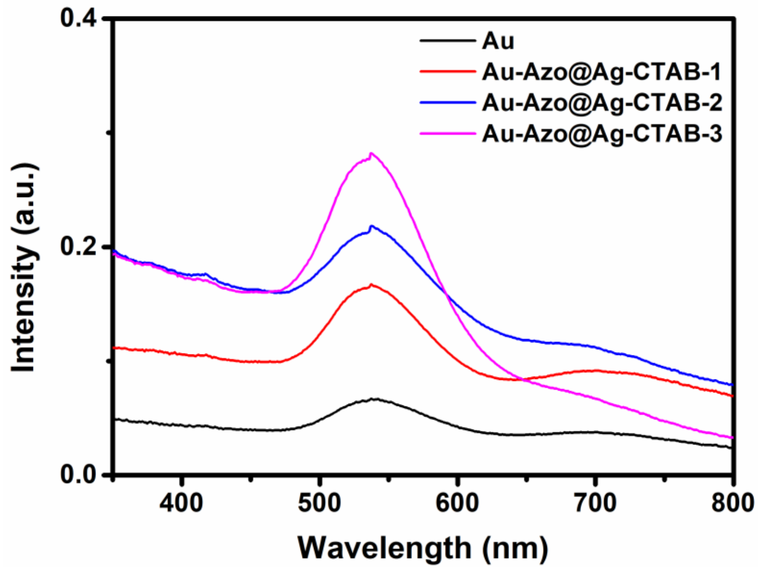

Figure S4. Optimization of the addition volume of CTAB. UV-vis absorption spectra of different Au−Azo@Ag−CTAB NPs.

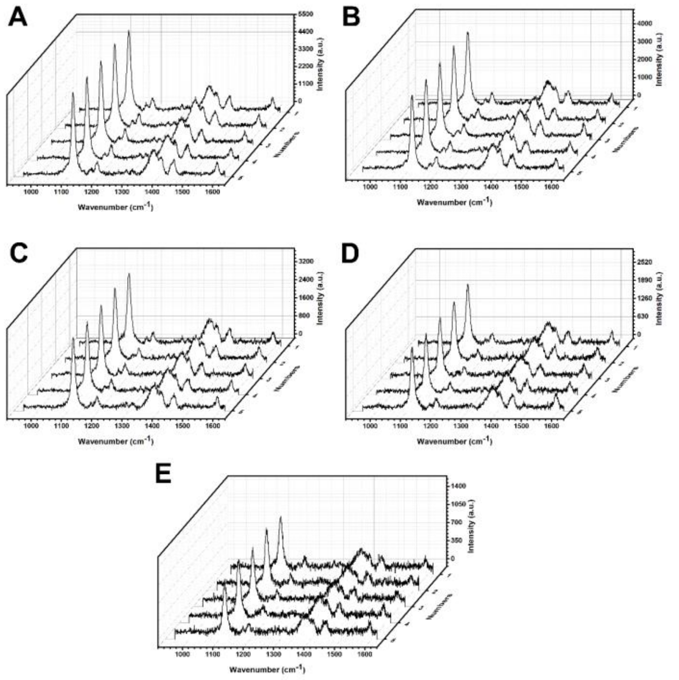

Figure S5. Raman spectra in five randomly selected positions of the capillary tube containing mixture of different concentrations of *E. coli* and nanotags. A: 10^7 CFU/mL; B: 10^6 CFU/mL; C: 10^5 CFU/mL; D: 10^4 CFU/mL; E: 10^3 CFU/mL. The relative standard deviation (RSD) of each group was less than 5%.

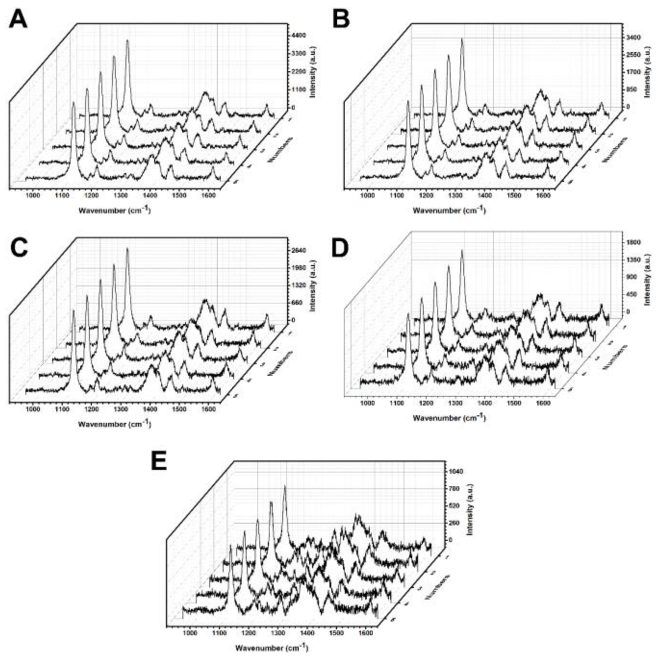

Figure S6. Raman spectra in five randomly selected positions of the capillary tube containing mixture of different concentrations of *S. aureus* and nanotags. A: 10^7 CFU/mL; B: 10^6 CFU/mL; C: 10^5 CFU/mL; D: 10^4 CFU/mL; E: 10^3 CFU/mL. The relative standard deviation (RSD) of each group was less than 5%.

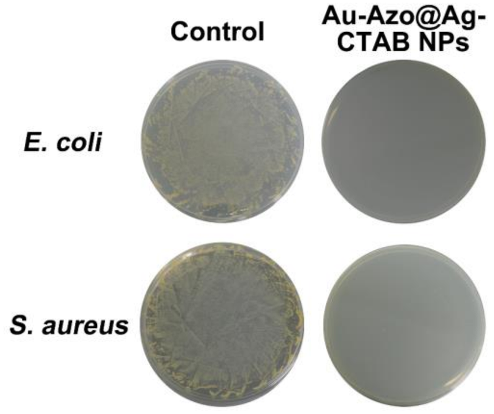

Figure S7. *E. coli* and *S. aureus* colonies after incubated with Au−Azo@Ag−CTAB nanotags for 2 h.
